# Association between urine pH and risk of contrast-associated acute kidney injury among patients after emergency percutaneous coronary intervention: a V-shape relationship?

**DOI:** 10.1007/s10157-020-02015-2

**Published:** 2021-01-11

**Authors:** Hanchuan Chen, Chen He, Zhebin You, Sicheng Zhang, Haoming He, Xi′nan Chen, Sunying Wang, Kaiyang Lin, Yansong Guo

**Affiliations:** 1 Fujian Provincial Key Laboratory of Cardiovascular Disease, Department of Cardiology, Fujian Provincial Hospital, Shengli Clinical Medical College of Fujian Medical University, Fuzhou, 350001 Fujian China; 2 Fujian Key Laboratory of Geriatrics, Department of Geriatric Medicine, Fujian Provincial Hospital, Fujian Provincial Center for Geriatrics, Fujian Medical University, Fuzhou, 350001 China

**Keywords:** Urine pH, Contrast-associated acute kidney injury, Emergency percutaneous coronary intervention

## Abstract

**Aim:**

We investigated whether perioperative urine pH was associated with contrast-associated acute kidney injury (CA-AKI) in patients undergoing emergency percutaneous coronary intervention (PCI).

**Methods:**

The study enrolled 1109 consecutive patients undergoing emergency PCI. Patients were divided into three groups based on perioperative urine pH (5.0–6.0, 6.5– 7.0, 7.5–8.5). The primary endpoint was the development of CA-AKI, defined as an absolute increase ≥ 0.3 mg/dL or a relative increase ≥ 50% from baseline serum creatinine within 48 h after contrast medium exposure.

**Results:**

Overall, 181 patients (16.3%) developed contrast-associated acute kidney injury. The incidences of CA-AKI in patients with urine pH 5.0–6.0, 6.5–7.0, and 7.5–8.5 were 19.7%, 9.8%, and 23.3%, respectively. After adjustment for potential confounding factors, perioperative urine pH 5.0–6.0 and 7.5–8.5 remained independently associated with CA-AKI [odds ratio (OR)1.86, 95% confidence interval (CI) 1.25–2.82, *P* = 0.003; OR 2.70, 95% CI 1.5–4.68, *P* < 0.001, respectively]. The association was consistent in subgroups of patients stratified by several CA-AKI risk predictors. However, the risk of CA-AKI associated with urine pH 7.5–8.5 was stronger in patients with worse renal function (estimated glomerular filtration rate (eGFR) < 60 mL/min/1.73m^2^) (HR 5.587, 95% CI 1.178–30.599 vs. HR 2.487, 95% CI 1.331–4.579; overall interaction *P* < 0.05).

**Conclusion:**

The urine pH and CA-AKI may underlie the V-shape relationship.

## Introduction

Contrast-associated acute kidney injury (CA-AKI), as a common complication after coronary intervention, is the third leading cause of hospital acquired acute kidney injury [1]. It increases length of hospitalization, medical expenses and risk of dialysis and major adverse cardiovascular events (MACE) [2].

Many strategies have been tested to prevent CA-AKI with more or less success, and hydration is recommended by current guidelines. Infusion of sodium bicarbonate instead of sodium chloride was ever advocated for urinary alkalization and reducing the generation of free radicals [3]. Some studies found administration of sodium bicarbonate was effective in preventing CA-AKI [4, 5], but some others drew different conclusions [6, 7], even harmful [8].

Recent studies showed urine pH was an independent risk factor of AKI [9, 10]. However, the predictive value of urine pH on the development of CA-AKI in patients undergoing emergency PCI remains unclear. Therefore, in current study, we sought to explore the relationship between urine pH and CA-AKI in patients undergoing emergency PCI.

## Materials and methods

### Patients

This is a prospective observational study conducted in Fujian Provincial Hospital in China, from January 2012 to December 2018. A total of 1210 consecutive patients undergoing emergency PCI were enrolled. Exclusion criteria were as follows: (1) lack of data on pre-procedural or post-procedural serum creatinine (SCr) levels (*n* = 60); lack of data on perioperative urine pH (*n* = 20); cancer with expectation of life less than 1 year (*n* = 6); end-stage renal disease (eGFR < 15 mL/min/1.73m^2^) or long-term dialysis treatment (*n* = 6); intra-vascular administration of contrast medium within the last 7 days postoperatively (*n* = 5); died within 24 h after admission (*n* = 4). Finally, 1109 patients were included in the analysis. The study was approved by an institutional review committee and the subjects gave informed consent.

### Protocol

The concentration of urine pH was measured for each patient during the perioperative period of PCI. SCr was measured at admission and daily for the 2 days after contrast exposure. We also measured white blood cell count (WBC), platelet (PLT), hemoglobin, cholesterol, glucose concentrations (GLU), troponin I, N-terminal pro-B-type natriuretic peptide (NT-pro-BNP) and other parameters before PCI procedure. Experienced interventional cardiologists performed PCI and used medications according to standard clinical practice. Nonionic, low-osmolar contrast media (either Iopamiron or Ultravist, both 370 mg I/mL) was used in procedural and 0.9% normal saline (NS) at a rate of 1 mL/kg/h was administered intravenously approximately 12 h during perioperative period (0.5 mL/kg/h if patients with heart failure). The protocol fulfilled the requirements of the Declaration of Helsinki and was approved by the ethics committee of the Fujian Provincial Hospital, China (ethics approval number: K2019-07-011).

### Definitions and follow-up

The primary endpoint was CA-AKI, defined as an absolute increase ≥ 0.3 mg/dL or a relative increase ≥ 50% from baseline serum creatinine within 48 h of contrast medium exposure [11, 12]. Additional end points were long-term mortality. The eGFR was calculated using the modified modification of diet in renal disease equation: 186.3 × SCr-1.154 × (age in years) -0.203 × 1.212 (if patient was black) × 0.742 (if patient was female) [13]. Peri-hypotension was defined as within 24-h periprocedural period, systolic blood pressure (SBP) < 80 mmHg for at least 1 h and medications or intra-aortic balloon pump (IABP) was needed [14]. All patients were followed for more than 1 year. Follow-up events were carefully monitored and recorded by trained nurses through outpatient visits or post-discharge telephone contacts with patients or their relatives.

### Statistical analysis

All data were analyzed with R version 4.0.2. We compared the baseline characteristics among 2 groups divided by CA-AKI. Normally distributed continuous variables are expressed as mean ± standard deviation (SD). The Student’s *t* test, Wilcoxon rank sum test or one way-analysis of variance was performed to determine the differences among groups. Categorical variables were compared by Chi-square test or Fisher exact test. The *P* for trend was determined with a Wilcoxon type test for trend across ordered groups. Kaplan–Meier curve were used to compute the cumulative incidence of mortality stratified by urine pH levels.

After testing for proportional hazard assumptions, logistic analysis was used to examine the association of urine pH 5.0–6.0 and urine pH 7.5–8.5 (vs. urine pH 6.5–7.0) with CA-AKI in models adjusted as follows: model 1 adjusted for traditional risk factors for CA-AKI (age > 75 years, diabetes mellitus, eGFR < 60 mL/min/1.73m^2^, and LVEF < 40%); and model 2 adjusted for variables in model 1 plus the variables with *P* value < 0.05 in the univariate statistical results including atrial fibrillation (AF), perioperative hypotension, LgNT-pro-BNP, GLU, WBC. Subgroup analysis in the study participants stratified by the several CA-AKI risk factors was also examined and the *P* values for interaction were calculated in each subgroup. A 2-sided *P* value < 0.05 was considered significant.

## Results

### Baseline characteristics

This study included 1109 consecutive patients, of whom 181 (16.3%) developed CA-AKI. Table 1 shows the univariate analysis of the baseline and procedural characteristics between the patients with and without CA-AKI. Patients who developed CA-AKI were older, more likely to have AF and worse renal function, had higher baseline of NT-pro-BNP, WBC, GLU, and had a higher percentage of perioperative hypotension, but lower left ventricular ejection fraction (LVEF).

**Table 1:** Baseline variables between non-AKI group and AKI group

	Non-AKI	AKI	*P* value
	(*n* = 928)	(*n* = 181)	
Demographics			
Age, years	62.46 ± 12.20	66.60 ± 12.46	< 0.001
Age > 75 years [*n* (%)]	156 (16.8)	53 (29.3)	< 0.001
Sex, female [*n* (%)]	126 (13.6)	33 (18.2)	0.129
Systolic blood pressure (mmHg)	123.01 ± 22.49	118.96 ± 27.53	0.065
Diastolic blood pressure (mmHg)	73.41 ± 15.55	71.35 ± 19.27	0.179
Medical history			
Hypertension [*n* (%)]	531 (57.2)	116 (64.1)	0.103
Type of ACS			0.254
STEMI [*n* (%)]	775 (83.5)	160 (88.4)	
NSTEMI [*n* (%)]	111 (12.0)	15 (8.3)	
UA, *n* (%) [*n* (%)]	42 (4.5)	6 (3.3)	
Diabetes [*n* (%)]	324 (34.9)	79 (43.7)	0.032
Atrial fibrillation [*n* (%)]	53 (5.7)	32 (17.7)	< 0.001
Anemia [*n* (%)]	211 (22.7)	40 (22.1)	0.928
Medical therapy during hospitalization			
Statin use [*n* (%)]	920 (99.1)	178 (98.3)	0.401
CCB use [*n* (%)]	113 (12.2)	23 (12.7)	0.940
Antiplatelet agents use [*n* (%)]	891 (96.0)	168 (92.8)	0.089
Laboratory measurements			
NT-proBNP (pg/mL)	1095.96 ± 2777.96	2360.510 ± 4711.11	0.001
WBC (10^9^/L)	11.36 ± 3.79	12.78 ± 4.57	< 0.001
HGB (g/L)	141.75 ± 18.09	140.25 ± 19.47	0.339
PLT (10^12^/L)	228.11 ± 69.88	226.39 ± 59.65	0.733
Cholesterol (mmol/L)	4.8 0 ± 1.21	4.72 ± 1.22	0.457
LDL-C(mmol/L)	3.24 ± 1.06	3.15 ± 1.05	0.343
eGFR (mL/min/1.73 m^2^)	100.37 ± 28.90	92.34 ± 36.24	0.005
eGFR < 60 mL/min/1.73 m^2^ [*n* (%)]	63 (6.8)	31 (17.1%)	< 0.001
LVEF	55.10 ± 7.10	49.65 ± 9.91	< 0.001
LVEF < 40% [*n* (%)]	28 (3.0%)	23 (12.7)	< 0.001
Procedure performed			
Perioperative hypotension [*n* (%)]	235 (25.3)	77 (42.5)	< 0.001
Contrast volume (mL)	168.83 ± 47.18	175.54 ± 49.24	0.093
Iso-osmolar contrast media use [*n* (%)]	294 (31.7)	49 (27.1)	0.255

### Risk factors of CA-AKI

**Fig. 1:**
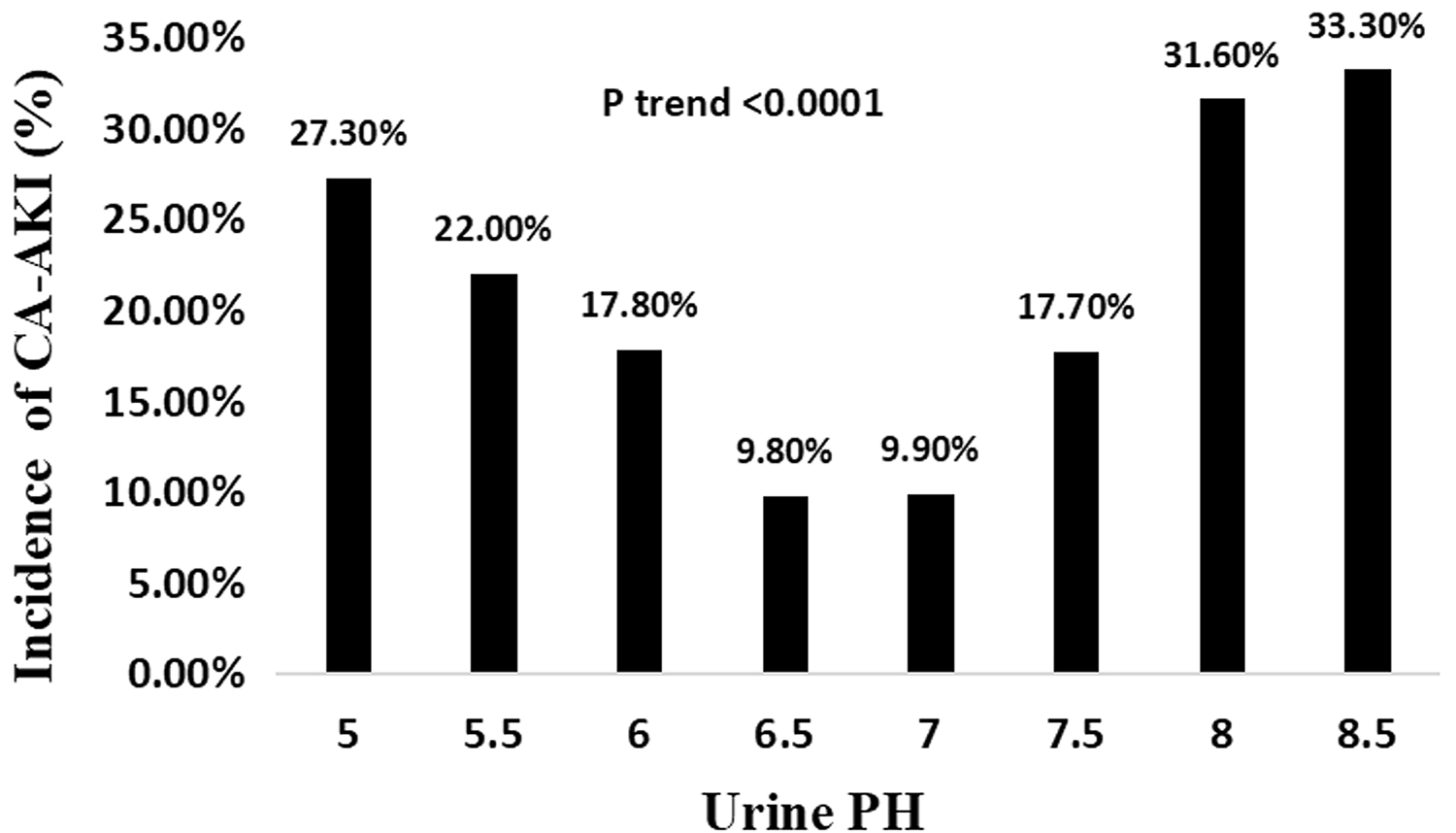
Incidence of CA-AKI

The incidence of CA-AKI was 27.30%, 22.00%, 17.80%, 9.80%, 9.90%, 17.70%, 31.60% and 33.30% in patients with perioperative urine pH 5, 5.5, 6, 6.5, 7, 7.5, 8, 8.5, respectively (Fig. 1). In multivariable-adjusted logistic proportional hazard models, compared with urine pH 6.5–7.0, urine pH 5.0–6.0 and urine pH 7.5–8.5 were both significantly associated with increased risk of CA-AKI, independent of demographics and clinical risk factors (Table 2). In model 1, after adjusted for traditional risk factors including age > 75 years, diabetes mellitus, eGFR < 60 mL/min/1.73 m^2^, and LVEF < 40%, perioperative urine pH 5.0–6.0 and 7.5–8.5 were both significantly correlated with CA-AKI, and the odds ratios (OR) values were 2.03 (95% CI 1.38–3.02, *P* < 0.001) and 2.62 (95% CI 1.53–4.45, *P* < 0.001), respectively. In model 2, after adjusting for variables in model 1 plus other variables including AF, perioperative hypotension, LgNT-pro-BNP, GLU and WBC, perioperative urine pH 5.0–6.0 and 7.5–8.5 remained associated with increased risk of CA-AKI, the OR values were 1.86 (95% CI 1.25–2.82, *P* = 0.003) and 2.70 (95% CI 1.55–4.68, *P* < 0.001), respectively.

**Table 2:** Associations between urine pH levels and CA-AKI

		Participants (*n*)	Events (*n*)	Rate (%)	Model 1^a^ OR (95% CI)	*P* value	Model 2^b^ OR (95% CI)	*P* value
Urine PH	6.5–7	427	42	9.8	1.00 (Ref.)		1.00 (Ref.)	
	5–6	553	109	19.7	2.03 (1.38–3.02)	< 0.0001	1.86 (1.25–2.82)	0.003
	7.5–8.5	129	30	23.3	2.62 (1.53–4.45)	< 0.0001	2.70 (1.55–4.68)	< 0.0001

*CI* confidence interval, *HR* hazard ratio

^a^Model 1 adjusted for age > 75 years, diabetes mellitus, eGFR < 60 mL/min/1.73m^2^, and LVEF < 40%

^b^Model 2 adjusted for variables in model 1 plus atrial fibrillation, perioperative hypotension, LgNT-pro-BNP, GLU, WBC

### Subgroup analysis based on CA-AKI risk predictors

**Fig. 2:**
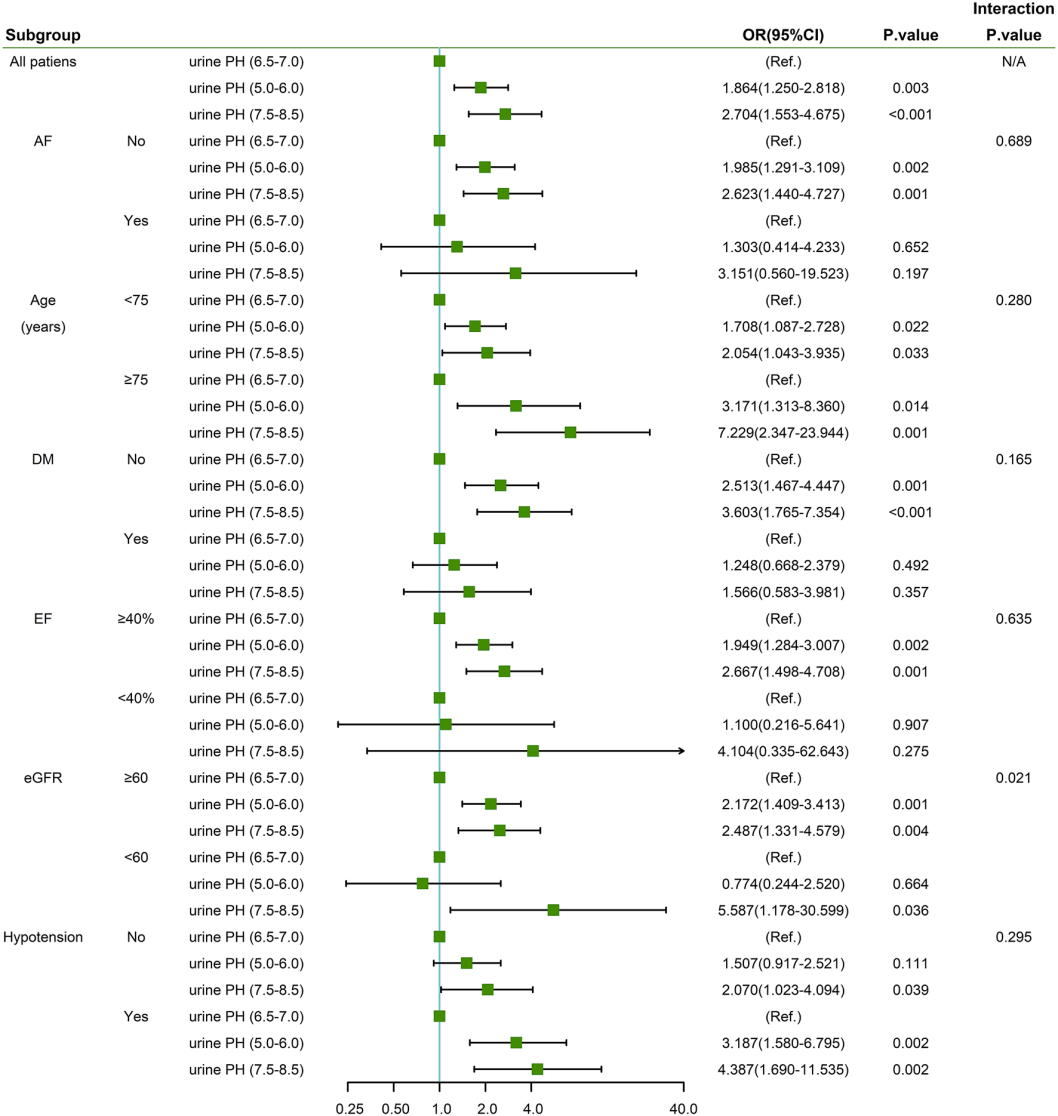
Forest and interaction

Figure 2 shows subgroup analyses stratified by several CA-AKI risk predictors. The associations between urine pH and CA-AKI are consistent among these subgroups. There was no effect modification of age > 75 years, diabetes mellitus, hypertension, AF or LVEF < 40% on the association between urine pH and risk of CA-AKI. However, there was an effect modification by eGFR: the risk of CA-AKI associated with urine pH 7.5–8.5 was stronger in patients with worse renal function (eGFR < 60 mL/min/1.73 m^2^) than in those with eGFR≧60 mL/min/1.73 m^2^. (HR 5.587, 95% CI 1.178–30.599 vs. HR 2.487, 95% CI 1.331–4.579; overall interaction *P* < 0.05).

### CA-AKI, urine pH level and long-term outcomes

**Fig. 3:**
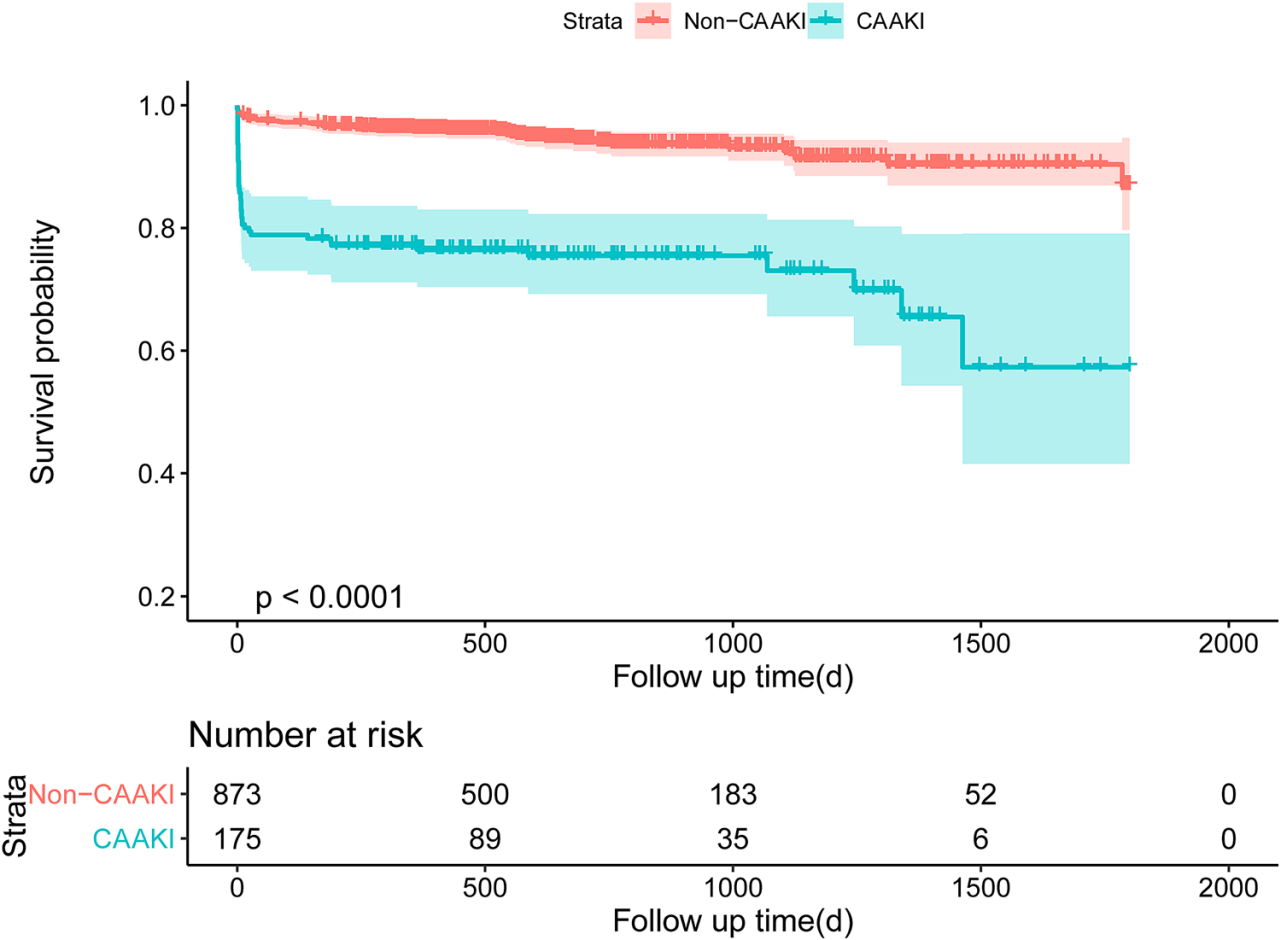
Mortality between CA-AKI

**Fig. 4:**
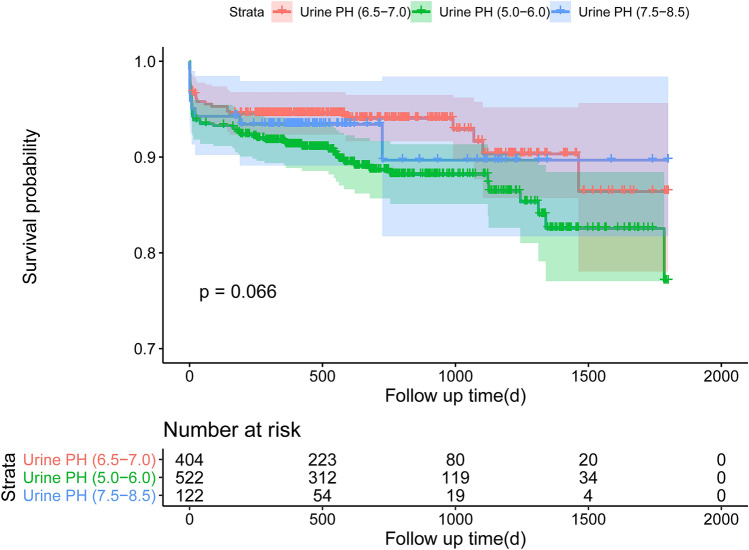
Mortality between urine pH

The median follow-up period was 559 days (interquartile range 371–912 days). Compared with patients without CA-AKI, the Kaplan–Meier curve showed that patients with CA-AKI had higher rate of all-cause long-term mortality (*P* < 0.001) (Fig. 3). However, there was no significantly difference in all-cause long-term mortality among three different groups stratified by urine pH levels (Fig. 4).

## Discussion

To our knowledge, this study is first to observe a V-shape relationship between urine pH and CA-AKI. Perioperative urine pH was found to be associated with the development of CA-AKI. Compared with the relatively normal urine pH value (between 6.5 and 7), both acid urine (5–6) and alkaline urine (7.5–8.5) were significantly associated with increased risk of CA-AKI.

CA-AKI, as the third leading cause for hospital acquired AKI, was known to increase the co-morbidities, in-hospital mortality and length of hospitalization [5]. Due to various factors including hemodynamic instability, insufficient hydration and cardiac dysfunction, the risk of CA-AKI was higher in patients undergoing emergency PCI than those undergoing elective PCI [15]. In addition to hydration and contrast agent dose reduction, pharmacological strategies such as furosemide [8], *N*-acetylcysteine [9], statin [10], ascorbic acid [11] and sodium bicarbonate [4, 12] were also evaluated. However, no effective treatment for CA-AKI in high-risk patients has been defined.

Sodium bicarbonate was supposed to be useful in the prevention of CA-AKI for urine alkalization and reducing the creation of free oxygen radicals. However, the results were not consistent.

In 2004, Gregory et al. [4] first proved the effectiveness of preventing contrast-associated renal failure by hydration with sodium bicarbonate instead of normal saline(NS) before contrast exposure(13.6% vs. 1.7%, *P* = 0.02). Subsequently, some studies showed the intravenous sodium bicarbonate administration reduced the risk of CA-AKI in patients at medium to high risk [5] or undergoing emergency PCI [16, 17]. Furthermore, a meta-analysis [18] demonstrated that hydration with bicarbonate reduced the incidence of CA-AKI compared with NS. However, some later trials showed different results [7, 19, 20], and even suggested that bicarbonate may increase the risk of CA-AKI. A retrospective cohort study at Mayo Clinic [8] showed that urine alkalinization by bicarbonate alone was associated with increased risk of CA-AKI compared with placebo, and a prospective randomized trial conducted by Theresia Klima et al. [21] drew similar conclusion in patients with renal insufficiency. The inconsistency of the results may be associated with following factors: (1) urine alkalization was not achieved in all studies. (2) The definitions of CA-AKI were different in some studies. (3) Some studies included low-risk patients with intact baseline kidney function but some included patients with moderate-to-severe renal dysfunction [20, 22, 23]. In 2018, PRESERVE trial [24] included 4993 patients and showed there was no benefit of intravenous sodium bicarbonate over intravenous sodium chloride for the prevention of CA-AKI among patients at high risk for renal complications who were undergoing angiography. Urine alkalization was confirmed by the higher urine pH observed in the sodium bicarbonate group compared with sodium chloride group (6.7 ± 0.8 vs. 6.0 ± 0.8, *P* < 0.001). It seemed that there was no additional benefit of bicarbonate in preventing CA-AKI compared to sodium chloride.

However, previous studies including PRESERVE trial did not study the relationship between urine PH and CA-AKI. It is unclear whether the inconsistent conclusion of sodium bicarbonate in preventing CA-AKI was affected by different urine pH levels.

Our study first found a V-shape relationship between urine pH and CA-AKI in patients undergoing emergency PCI. The result showed both acid urine (5–6) and alkaline urine (7.5–8.5) were significantly associated with increased risk of CA-AKI. Recent studies have shown acid urine was an independent risk factor of CA-AKI. Darko Markota et al.[9] found patients with a urine pH < 6 had a more than tenfold higher risk of CA-AKI. Jun-qing Yang et al. also found [10] urine pH < 6 was associated with higher risk of CA-AKI in T2DM patients undergoing elective contrast media exposure. It may be explained by the increased level of free radical formation in an acid medium compared with the higher PH of normal extracellular fluid [25]. Our studies supported the urine pH < 6.5 was associated with higher risk of CA-AKI. More importantly, our study showed a higher urine pH (7.5–8.5) was also an independent risk factor of CA-AK, which was not reported in previous studies. Based on the V-shape relationship between urine pH and CA-AKI observed in our study, it is possible that excessive urine alkalization may promote renal injury, which may be the reason why previous trials drew different conclusions.

In addition, subgroup analysis showed the V-shape association between urine pH and CA-AKI was consistent in patients stratified by several CA-AKI risk predictors, especially in those with worse renal function. It suggested patients with renal insufficiency may have higher risk of CA-AKI when they had a higher or lower perioperative urine pH. However, it was CA-AKI not urine PH that associated with higher long-term mortality in our study, which need more large studies to confirm in the future.

The mechanism by which patients with neutral perioperative urine pH have minimum risk of CA-AKI is multifactorial. Within an acid environment-free radical formation is promoted for activated Harber–Weiss reaction (HWR), resulting in an increased renal oxidant injury and occurrence of CA-AKI [26, 27]. In addition, superoxide generated by ischemia might react with medullary nitric oxide to form the potent oxidant peroxynitrite [28]. At physiologic concentrations, bicarbonate scavenges reactive species especially peroxynitrite generated from nitric oxide [29]. On the other hand, a higher urine pH may reflect the dysfunction of renal tubular cells which secrete H + and reabsorb bicarbonate physiologically [30, 31]. Meanwhile, reactive oxygen species (ROS) like peroxymonocarbonate (HCO4-) can be activated by sodium bicarbonate as described by Richardson et al. [32], which reveals that excessive alkalization may promote renal injury.

### Limitations

There are several limitations in our study. First, this study was a single-center, observational study, which potentially may limit the generalizability of the findings. Second, for the restricted length of hospitalization in patients with emergency PCI, assessments for detecting CA-AKI in our study are limited to the first 48 h and some actual CA-AKI patients may be missed. Third, patients in our study were not routinely treated with sodium bicarbonate strategy. Fourth, data about blood gas analysis and volume of fluid administered before procedure are lacking, which may influence the incidence of CA-AKI. Fifth, data about AKI stage were limited in our study. Despite these limitations, our results provided useful insights into the correlation of baseline urine pH with the incidence of CA-AKI.

## Conclusion

In conclusion, urine pH showed a V-shape association with the risk of CA-AKI in patients undergoing emergency PCI. Excessive acid or alkaline urine was associated the increased risk of CA-AKI. As the test of urine pH is common and simple, it could be used to identify high-risk patients and guide the sodium bicarbonate strategy for preventing CA-AKI.
